# Schwann cells promote prevascularization and osteogenesis of tissue-engineered bone via bone marrow mesenchymal stem cell-derived endothelial cells

**DOI:** 10.1186/s13287-021-02433-3

**Published:** 2021-07-07

**Authors:** Xinxin Zhang, Xiaorui Jiang, Shan Jiang, Xiyu Cai, Shengji Yu, Guoxian Pei

**Affiliations:** 1 Department of Orthopaedics, National Cancer Center/National Clinical Research Center for Cancer/Cancer Hospital, Chinese Academy of Medical Sciences and Peking Union Medical College, No. 17 Panjiayuannanli, Chaoyang District, Beijing, 100021 China; 2 Department of Hand and Foot Orthopaedics, Yantai Yuhuangding Hospital, Qingdao University Medical College, Yantai, Shandong China; 3 Department of Clinical Medicine, Nanfang Hospital, Southern Medical University, Guangzhou, Guangdong China; 4 Department of Orthopedics, The Fifth Affiliated Hospital of Sun Yat-Sen University, Zhuhai, Guangdong China; 5 Southern University of Science and Technology Hospital, No. 6019 Liuxian Street, Xili Avenue, Nanshan District, Shenzhen, 518055 Guangdong China

**Keywords:** Schwann cells, Endothelial cells, Bone marrow-derived mesenchymal stem cells, Prevascularization, Bone tissue engineering, Nestin, TIMP-2

## Abstract

**Background:**

Tissue-engineered bone grafts (TEBGs) that undergo vascularization and neurotization evolve into functioning bone tissue. Previously, we verified that implanting sensory nerve tracts into TEBGs promoted osteogenesis. However, the precise mechanisms and interaction between seed cells were not explored. In this study, we hypothesized that neurotization may influence the osteogenesis of TEBGs through vascularization.

**Methods:**

We cultured rat Schwann cells (SCs), aortic endothelial cells (AECs), and bone marrow-derived mesenchymal stem cells (BM-MSCs) and then obtained BM-MSC-derived induced endothelial cells (IECs) and induced osteoblasts (IOBs). IECs and AECs were cultured in an SC-conditioned medium (SC-CM) to assess proliferation, migration, capillary-like tube formation, and angiogenesis, and the vascular endothelial growth factor (VEGF) levels in the supernatants were detected. We established an indirect coculture model to detect the expression of nestin and VEGF receptors in IECs and tissue inhibitor of metalloproteinase (TIMP)-2 in SCs. Then, SCs, IECs, and IOBs were labeled and loaded into a β-tricalcium phosphate scaffold to induce prevascularization, and the scaffold was implanted into a 6-mm-long defect of rat femurs. Three groups were set up according to the loaded cells: I, SCs, and IECs (coculture for 3 days) plus IOBs; II, IECs (culture for 3 days) plus IOBs; III, IOBs. Nestin and TIMP-2 expression and osteogenesis of TEBGs were evaluated at 12 weeks post-implantation through histological and radiological assessments.

**Results:**

We found that SC-CM promoted IEC proliferation, migration, capillary-like tube formation, and angiogenesis, but no similar effects were observed for AECs. IECs expressed nestin extensively, while AECs barely expressed nestin, and SC-CM promoted the VEGF secretion of IECs. In the coculture model, SCs promoted nestin and VEGF receptor expression in IECs, and IECs inhibited TIMP-2 expression in SCs. The promotion of prevascularized TEBGs by SCs and IECs in group I augmented new bone formation at 6 and 12 weeks. Nestin expression was higher in group I than in the other groups, while TIMP-2 expression was lower at 12 weeks.

**Conclusions:**

This study demonstrated that SCs can promote TEBG osteogenesis via IECs and further revealed the related specific characteristics of IECs, providing preliminary cytological evidence for neurotization of TEBGs.

**Supplementary Information:**

The online version contains supplementary material available at 10.1186/s13287-021-02433-3.

## Background

Although great progress has been made in the field of bone tissue engineering, large bone defects caused by trauma, infection, or tumors remain an unsolved issue. The nerves and blood vessels are both indispensable for normal bone. There is clear evidence that the efficiency of bone formation and homeostasis is dependent on the level of angiogenesis and vascularization [1]. The vasculature within a graft supplies oxygen and nutrients, removes metabolites, and provides specific hormones, growth factors, and neurotransmitters to cells seeded in the graft, allowing the successful transformation of tissue-engineered bone grafts (TEBGs) into functional bone tissues and thereby ensuring their survival and stimulating their activity [1, 2]. The microscopic architecture of compact tissues of bone is characterized by osteons and Haversian channels containing both nerves and blood supply [3]. The coexistence of interwoven microvasculature and functional nerve fibers in TEB contributes to the formation of physiologically mature bone. The distribution of nerves in bones was recognized very early [4, 5]. In physiological bone tissue, nerves are distributed throughout the periosteum, cortex, and marrow parenchyma and are associated with marrow blood vessels [6]. The nerves, blood vessels, and bones interact reciprocally and in a complex manner in tissue-engineered bone (TEB).

It has been confirmed that neuropeptides and neurotrophins secreted by nerve fibers within the musculoskeletal system affect bone [7]. Nerve fibers are able to regulate fundamental biological processes such as remodeling, metabolism, hematopoiesis, and angiogenesis in bone tissues [8–10]. Bone is innervated by sensory and sympathetic nerve fibers that, in addition to skeletal pain transmission, play a role in bone metabolism [3]. Many neuropeptides secreted by sensory and autonomic nerves within bone tissues, including substance P, calcitonin gene-related peptide from sensory nerves, vasoactive intestinal peptide from parasympathetic nerves, and neuropeptide Y from sympathetic nerves, positively regulate the function of bone cells [11–13]. Similarly, neurotrophins, including nerve growth factor (NGF) and brain-derived neurotrophic factor (BDNF), directly stimulate bone cells [14, 15].

Nerves affect the blood vessels in the process of TEB construction. Our previous studies concluded that implanting sensory nerve tracts into TEBGs can significantly enhance both vascularization and neurotization simultaneously to obtain a better osteogenic effect [16–18]. Schwann cells (SCs), as glial cells in peripheral nerves, can provide trophic support for axonal regeneration and neurogenesis and secrete a variety of neurotrophic factors, including NGF and BDNF. Previous studies have suggested that NGF and BDNF are able to promote angiogenesis [19–21]. However, some studies have shown that SC-derived tissue inhibitor of metalloproteinase (TIMP)-2 inhibits angiogenesis [22, 23]. The interaction between blood vessels and nerves during TEB osteogenesis is complicated.

We hypothesized that neurotization improves the performance of a TEGB by inducing angiogenesis and osteogenesis. To explore the interactions between neurotization and vascularization in TEB, it is imperative to understand the relationship between SCs and endothelial cells and the biological mechanisms that underlie the relationship. In this study, we aimed to reveal the relationship between SCs and endothelial cells during TEB construction and provide cytological evidence for simultaneous neurotization and vascularization of TEB.

## Methods

### Cell culture and induction

All animal experiments were approved by the Animal Welfare and Ethics Committee of Southern Medical University. Rat SCs were isolated from the sciatic nerves in SC regular medium by using tissue explants adherent method and purified at passage 2 in SC medium for purification, and aortic endothelial cells (AECs) were isolated from the intima of rat thoracic aortas by using collagenase digestion method, and bone marrow-derived mesenchymal stem cells (BM-MSCs) were isolated from the bone marrow of rat femurs and tibias by using density gradient centrifugation method according to previously described protocols [24–27]. SCs were purified through dissection of the epineurium and by using differential adhesion method for 2~3 times and cultured in SC medium for purification for 24 h to suppress the contaminated fibroblasts [24, 25]. AECs were purified through separating vascular intima from vascular adventitia and media to suppress the contaminated vascular smooth muscle cells and fibroblasts. BM-MSCs at passage 3 were differentiated into induced endothelial cells (IECs) or induced osteoblasts (IOBs) by corresponding induction medium for 21 days. The composition of the culture medium used is shown in Table 1.

**Table 1:** Composition of cell culture medium

Culture medium	Composition	Manufacturer’s name and location
SC regular medium	DMEM containing: 10% FBS 2 μM forskolin 10 ng/ml heregulin-β-1	HyClone, Thermo Fisher Scientific, Pittsburgh, PA Gibco, USA Gibco, Gaithersburg, MD BioVision, Milpitas, CA PeproTech, Rocky Hill, NJ
SC medium for purification	DMEM containing: 10% FBS 10 μM Ara-C	Hyclone Gibco Sigma-Aldrich, St. Louis, MO
AEC medium	M199 containing: 5% FBS 20 μg/ml ECGS 100 μg/ml heparin	Hyclone Gibco ScienCell, Carlsbad, CA Sigma-Aldrich
BM-MSC medium	DMEM containing: 10% FBS	Hyclone Gibco
IEC induction medium	DMEM (4.5 g/l glucose) containing: EGM-2 SingleQuots	Hyclone Lonza, Basel, Switzerland
IOB induction medium	DMEM (4.5 g/l glucose) containing: 10 nM dexamethasone 50 μg/ml ascorbic acid 10 mM sodium β-glyceryl phosphate	Hyclone Sigma-Aldrich Sigma-Aldrich Sigma-Aldrich

### Cell identification

SCs were characterized by immunocytochemistry staining for S100 and immunofluorescence staining for glial fibrillary acidic protein (GFAP), SRY-related HMG-box 10 (SOX10), myelin protein zero (MPZ), and growth-associated protein-43 (GAP43). AECs were characterized by immunocytochemistry staining for Factor VIII and immunofluorescence staining for Pecam1 and von Willebrand factor (vWF). A rabbit monoclonal antibody against Factor VIII (1:1000; Abcam, Cambridge, MA) and a mouse monoclonal antibody against S100 (1:200; Abcam) were used, and the nuclei were counterstained with hematoxylin. A rabbit monoclonal antibody against Pecam1 (1:100; Abcam), a rabbit monoclonal antibody against GFAP (1:250; Abcam), a rabbit polyclonal antibody against MPZ (1:100; Proteintech, Rosemont, IL), and a DyLight 488-conjugated secondary antibody (1:200; Abcam) were used, and the nuclei were then stained with 4′,6-diamidino-2-phenylindole (DAPI; Beyotime, Shanghai, China). A rabbit monoclonal antibody against SOX10 (1:25; Abcam), a rabbit monoclonal antibody against GAP43 (1:160; Abcam), a rabbit polyclonal antibody against vWF (1:400; Abcam), and a DyLight 594-conjugated secondary antibody (1:200; Abcam) were used, and the nuclei were then stained with DAPI. Classic BM-MSC markers, including CD90, CD29, and CD73, were detected by using flow cytometry and corresponding antibodies (Sungene Biotech, Tianjin, China). Changes in the morphology and organelles of BM-MSCs and IECs were observed by light microscopy and transmission electron microscopy (TEM). IECs were characterized by immunofluorescence staining for vWF and Pecam1. The antibody against vWF (1:400) and a rabbit monoclonal antibody against Pecam1 (1:100; Abcam) were used, and the nuclei were stained with DAPI. IOBs were identified by staining with alkaline phosphatase (ALP; Beyotime) and alizarin red S (Sigma-Aldrich).

### Conditioned medium

Confluent SCs in 10-cm dishes were rinsed three times with phosphate-buffered saline (PBS), and 10 ml of basal medium (high-glucose DMEM or M199) without FBS was added. This medium was harvested after 48 h and centrifuged for 5 min at 3000×*g*. Then, the supernatant was collected as SC-conditioned medium (CM)-DMEM or SC-CM-M199 and stored at − 70 °C until use.

### Cell proliferation assay

The proliferation of IECs or AECs in SC-CM-DMEM or SC-CM-M199 supplemented with 2% FBS (for IECs) or 5% FBS (for AECs) without other supplements was detected by using the CyQUANT Kit (Thermo Fisher Scientific) according to the manufacturer’s instructions. In the control groups, the basal medium was DMEM or M199. The original number of cells in each well of a 96-well plate was 1.5 × 10^3^ for IECs or 2.5 × 10^3^ for AECs. The fluorescence intensity in triplicate wells of each group was measured on days 1, 3, 5, 7, 9, and 11 at an excitation wavelength of 485 nm and an emission wavelength of 530 nm and converted to cell number according to a standard curve.

### Cell migration assay

An endothelial cell migration assay was performed by using Transwell chambers (pore size of 8 μm, 6.5 mm in diameter; Millipore, Billerica, MA). In quadruplicate, IECs or AECs (1.2 × 10^4^ cells/well) were seeded into the upper chambers in 120 μl of DMEM or M199 without FBS or other supplements, and 500 μl of SC-CM-DMEM or SC-CM-M199 (DMEM or M199 as a control) containing 0.5% bovine serum albumin (BSA; Beyotime) and 1% FBS was added to the lower chambers. The upper chambers were removed from the lower chambers after incubation at 37 °C for 6 h and wiped with cotton swabs. The polycarbonate membranes were fixed using methanol and stained with crystal violet. The membranes were eluted by using 100 μl of 10% acetic acid. The optical density (OD) of the eluted fluid in triplicate wells was measured at an absorbance of 570 nm.

### Capillary-like tube formation assay

Capillary-like tube formation was detected by using Matrigel (BD Biosciences, San Jose, CA). Liquid Matrigel was placed in a 96-well plate (60 μl/well). IECs or AECs (2 × 10^4^ cells/well) were seeded on Matrigel in 40 μl of SC-CM-DMEM or SC-CM-M199. After incubation for 4 h at 37 °C, phase contrast micrographs were taken. Capillary-like meshes were quantified by the ImageJ 1.4.3 software (National Institutes of Health, USA) in random fields in triplicate wells.

### Corneal angiogenesis assay

IECs were resuspended in concentrated SC-CM-DMEM or DMEM at a density of 1 × 10^7^ cells/ml. A total of 2 μl of medium containing cells was homogeneously mixed with 2 μl of 1% gelatin (Aladdin, Shanghai, China). The mixture was injected into the corneas of eight anesthetized 8-week-old female Sprague-Dawley (SD) rats according to the groups (4 rats per group), which were purchased from the Experimental Center of Southern Medical University (Guangzhou, China). After 12 days, the rats were sacrificed and perfused with waterproof drawing ink (Zhonghua, Shanghai, China) by intracardiac injection. The eyes were fixed with 10% neutralized buffered formalin, and then the corneas were excised to observe angiogenesis. Vascular segments were quantified by the ImageJ 1.4.3 software in random fields in quadruplicate samples.

### Indirect coculture of SCs and IECs

SCs and IECs were indirectly cocultured by using Transwell chambers (pore size of 0.4 μm, 24 mm in diameter; Millipore). SCs (1 × 10^4^ cells/well) were seeded into the upper chambers in 1.5 ml of DMEM supplemented with 10% FBS without EGM-2 SingleQuots. IECs (1 × 10^4^ cells/well) were seeded into the lower chambers in 2.6 ml of the same medium. IECs not cocultured with SCs were plated in the control wells. IECs were collected on days 3 and 7 to examine the effect of SCs on them.

Likewise, IECs (1 × 10^4^ cells/well) were seeded into the upper chambers in 1.5 ml of DMEM supplemented with 10% FBS without other supplements. SCs (1 × 10^4^ cells/well) were seeded into the lower chambers in 2.6 ml of the same medium. SCs not cocultured with IECs were plated in the control wells. SCs were collected on days 3 and 7 to examine the effect of IECs on them.

### Quantitative reverse transcription polymerase chain reaction (qPCR)

Total RNA was extracted from IECs, SCs, and BM-MSCs by using an RNeasy Mini Kit (Qiagen, Valencia, CA) with on-column DNA digestion to eliminate genomic contamination. cDNA was synthesized by using the iScript cDNA Synthesis Kit (Bio-Rad Laboratories, Hercules, CA). Real-time PCR was conducted by using SYBR Green PCR Master Mix (Applied Biosystems, Thermo Fisher Scientific). The primer sequences used are shown in Table 2. The fold changes in expression were calculated using the 2−ΔΔCt method [28].

**Table 2:** Primer sequences used for qPCR

Gene	Primer sequence forward 5′–3′	Primer sequence reverse 5′–3′
Pecam1	TTGGCACCATGAACAAACTAGCA	CGCTTCGGAGACTGGTCACA
CD34	TGCCGTCTGTCAATGTTTCTGATTA	TCGGATTCCTGAACATTTGATGTC
Kdr	AATGCCCATGACCAAGAATGTG	GGATAGAGCCGCGTGTCTGAA
Nos 2	CTCACTGTGGCTGTGGTCACCTA	GGGTCTTCGGGCTTCAGGTTA
Nos 3	GCGGCTGGTACATGAGTTCAGA	AGATCCATGCAGACAGCCACA
TIMP-2	GACACGCTTAGCATCACCCAGA	CTGTGACCCAGTCCATCCAGAG
MMP-14	GAGAACTTCGTGTTGCCTGATGAC	TTCTGGGCTTATCTGGGACAGAG
GAPDH	GGACCAGGTTGTCTCCTGTG	CACCTGGAGTACCGGATGT

### Enzyme-linked immunosorbent assay (ELISA)

Secreted vascular endothelial growth factor (VEGF) levels were detected with a Quantikine® rat VEGF assay (R&D Systems, Minneapolis, MN) according to the manufacturer’s instructions. VEGF concentration in the SC-CM-DMEM was detected firstly. IECs were cultured in SC-CM-DMEM or DMEM with 2% FBS. The VEGF concentration in the supernatants of the wells was detected by ELISA on days 1, 3, 5, and 7. The medium in all the detected wells was fully replaced 24 h before each time point at which the supernatant was collected and analyzed.

### Western blot (WB) analysis

Cells were harvested and lysed in radioimmunoprecipitation assay (RIPA) lysis buffer (Beyotime) to extract the protein. The supernatants collected from the coculture system or the wells without coculture on days 3 and 7 were concentrated after constant volume, and the protein of supernatants was obtained. Equivalent amounts of protein were separated on a sodium dodecyl sulfate (SDS) gel (Beyotime) and electrotransferred to the PVDF membranes (Millipore). WB analysis was performed using the appropriate diluted antibodies. Signals were detected using an ImageQuant LAS System (GE Healthcare Life Sciences, Marlborough, MA). Primary antibodies specific for Flt1, Kdr, nestin (Rat-401) (all from Santa Cruz Biotechnology, Santa Cruz, CA), and TIMP-2 (Thermo Fisher Scientific) and a control antibody specific for β-actin (Sigma-Aldrich) were used in this experiment.

### Immunofluorescence staining

IECs or AECs were fixed and incubated with a mouse monoclonal antibody against nestin (1:200). Then, they were rinsed and incubated with a secondary antibody conjugated to DyLight 488 (1:200). The nuclei were then stained with DAPI.

IECs cultured in SC-CM-DMEM for 7 days were first immunostained for nestin (1:200) as described above and then immunostained for Flt1 (1:200) following the same procedure. Different secondary antibodies, including the DyLight 488-conjugated antibody (1:200) for nestin and a DyLight 405-conjugated antibody (1:200, Abcam) for Flt1, were used. The cells were finally visualized under a TCS-SP5 confocal laser scanning microscope (Leica, Buffalo Grove, IL).

### Cell transfection and labeling

BM-MSCs at passage 3 were infected with lentiviral medium (GeneChem, Shanghai, China) containing enhanced green fluorescent protein (EGFP) or red fluorescent protein (RFP) according to the manufacturer’s instructions to obtain BM-MSCs expressing EGFP or RFP, which were called BM-MSCs-EGFP or BM-MSCs-RFP, respectively. Stably infected BM-MSCs were selected with 1.5 μg/ml puromycin (Solarbio, Beijing, China). BM-MSCs-EGFP were differentiated into IOBs, and BM-MSCs-RFP were differentiated into IECs for scaffold loading. SC nuclei were stained with 100 μg/ml Hoechst 33342 (Beyotime) to obtain labeled SCs.

### Preparation of scaffolds

Sterile cylindrical β-tricalcium phosphate (β-TCP) scaffolds (6 mm in length and 4 mm in diameter; pore size of 200~300 μm, porosity volume of > 85%) were obtained from Bio-lu Biomaterials Company (Shanghai, China). 3-(4,5-Dimethylthiazol-2-yl)-5-(3-carboxymethoxyphenyl)-2-(4-sulfophenyl)-2H-tetrazolium (MTS) Cell Proliferation Colorimetric Assay Kit (BioVision) was used to detect BM-MSC proliferation in scaffold extract fluid or regular DMEM according to the manufacturer’s instructions. The MTS reagent was injected directly into the scaffold loaded with BM-MSCs to verify the cell viability on the scaffold. The scaffolds were presoaked in DMEM overnight first. In group I, labeled SCs and IECs-RFP in 200 μl of DMEM (1:1 ratio of cell number) were loaded into scaffolds at a concentration of 2 × 10^7^ cells/ml using the negative pressure suction method. After 4 h, an additional 10 ml of DMEM was added to each well to completely submerge the scaffolds in the medium. The same number of IOBs-EGFP in 200 μl of DMEM was loaded into scaffolds that had been incubated for 3 days. After 4 h, the scaffolds were incubated in DMEM for another 3 days. In group II, only IECs-RFP were loaded in the first 3 days, and other procedures were the same as those in group I. In group III, only IOBs-EGFP were loaded and incubated for 3 days.

### Animal models

Thirty-six 10-week-old male SD rats were purchased from the Experimental Center of Southern Medical University and divided into three groups according to the scaffold groups described above (12 rats per group). The rats were anesthetized and sterilized. The left middle femur was exposed in the intermuscular space through a lateral longitudinal incision. Four holes were drilled in the outer side of the femoral shaft to allow two screws to be placed on either side of the proposed osteotomy site. After periosteal dissection, a 6-mm-long defect was created by using a scroll saw. A scaffold loaded with cells was implanted into the defect. A stainless steel linear reconstruction plate with four holes was then placed along the femur and fixed with four cortical screws. A 20-gauge steel wire was looped between the two screws on either side of the plate to strengthen fixation. The scaffold was fixed to the steel plate with two sutures to keep it stable. The subcutaneous and skin layers were closed in a routine manner.

### Radiological observations

We randomly selected six rats from each group for radiological examination at 6 weeks and 12 weeks. The conditions of X-ray examination included a voltage of 40 kV, a current of 50 mA, and an exposure time of 200 ms. After the steel plate and screws were removed, micro-CT scans were performed at 12 weeks with an X-ray tube voltage of 80 kV, a current of 500 μA, and an exposure time of 200 ms. The average bone volume/total volume (BV/TV %) values of the femoral defect areas were analyzed for each group.

### Histological assessments

After removing the steel plate and screws at 12 weeks, we obtained femur tissue samples from each group, embedded the samples in paraffin, and sectioned scaffold segments. The sections were deparaffinized and blocked, and immunohistochemistry (IHC) staining was performed at 6 weeks with anti-nestin (1:500) and anti-TIMP-2 (1:200) primary antibodies and corresponding secondary antibodies. H&E and Masson staining were performed at postoperative 12 weeks according to routine procedures.

### Statistical analysis

The data obtained are presented as the means ± SDs. The data from the cell and animal model experiments were analyzed using Student’s t test (parametric data) or the Mann-Whitney test (nonparametric data). The GraphPad Prism 8.0.1 software (GraphPad Software Inc., San Diego, CA) was used to perform calculations and generate graphs. *P* < 0.05 was considered statistically significant (***P < 0.001, **P < 0.01, and *P < 0.05; ns = nonsignificant).

## Results

### Identification of cells

**Fig. 1:**
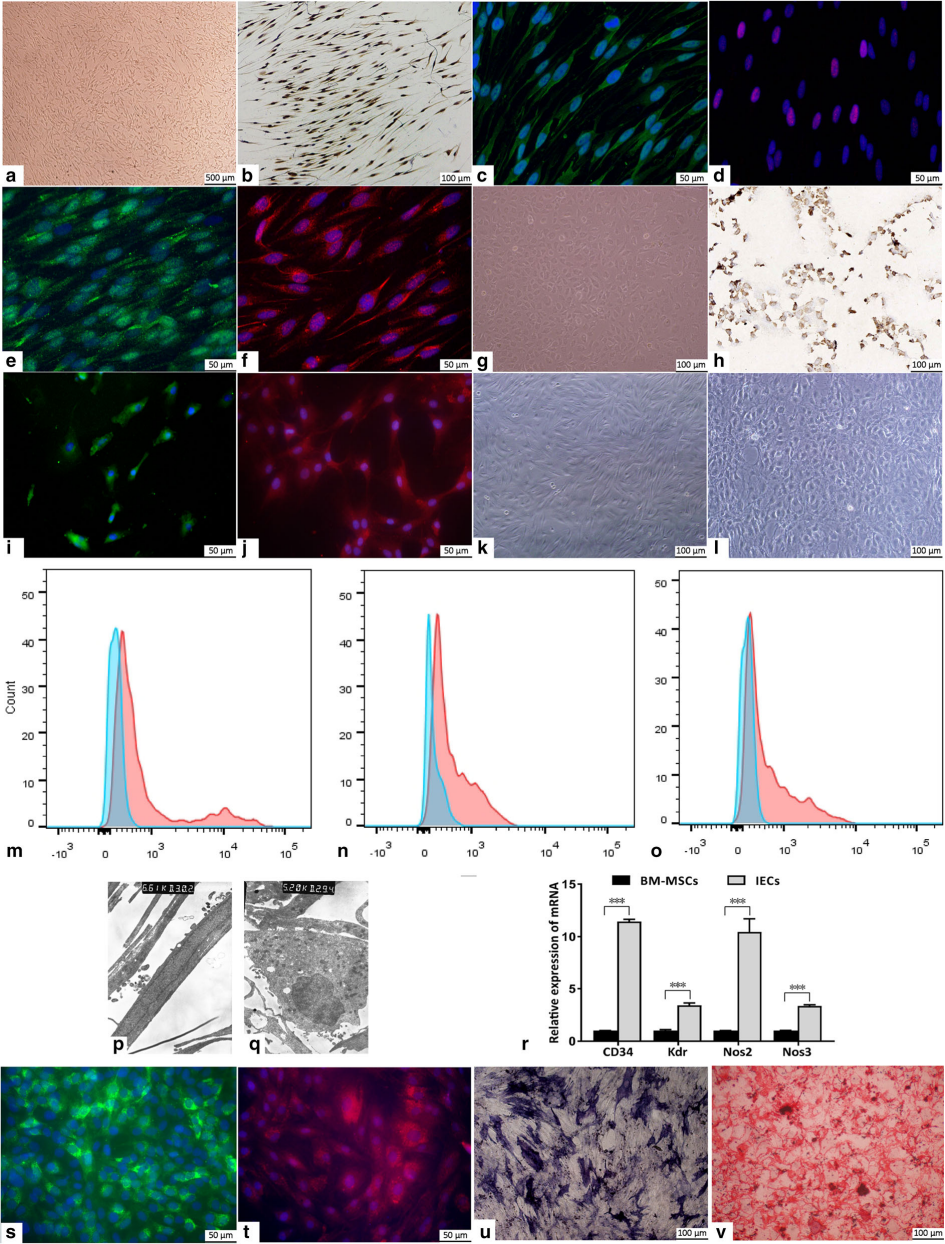
Cell culture and identification. **a** SCs at passage 2. **b–f** S100 (**b**), GFAP (**c**), SOX10 (**d**), MPZ (**e**), and GAP43 (**f**) immunocytochemical or immunofluorescence staining of SCs. **g** AECs at passage 2. **h** Factor VIII immunocytochemical staining of AECs. **i** Pecam-1 immunofluorescence staining of AECs. **j** vWF immunofluorescence staining of AECs. **k** BM-MSCs at passage 3. **l** IECs after induction for 21 days. **m–o** Flow cytometric detection of CD90 (**m**), CD29 (**n**), and CD73 (**o**) on IEC membrane (blue: isotype negative control, red: IECs). **p** TEM image of BM-MSCs (× 6610). **q** TEM image of IECs after induction for 21 days (× 5200). **r** CD34, Kdr, Nos2, and Nos3 mRNA expression levels in BM-MSCs and IECs, as assessed via real-time qPCR. **s** Pecam-1 immunofluorescence staining of IECs. **t** vWF immunofluorescence staining of IECs. **u, v** ALP (**u**) and alizarin red S (**v**) staining of IOBs after induction for 21 days

To identify SCs, AECs, BM-MSCs, IECs, and IOBs, we performed immunocytochemistry or immunofluorescence staining and another specific staining. The cytoplasm of SCs was positively stained for S100, GFAP, and GAP43; the cytoplasm and cytomembrane were positively stained for MPZ; and the nucleus was positively stained for SOX10 (Fig. 1a–f). The cytomembrane of AECs was positively stained for Factor VIII and Pecam1, and the cytomembrane and cytoplasm of AECs were positively stained for vWF (Fig. 1g–j). The morphology of the IECs changed from the fibrous spindle appearance of BM-MSCs to a cobblestone-like morphology (Fig. 1k, l). IECs displayed negative status or negative tendency for classic MSC markers, including CD90, CD29, and CD73 (Fig. 1m–o). The TEM results showed that IECs had more synapses, organelles, and phagocytic vesicles than BM-MSCs and that Weibel-Palade (W-P) bodies were observed near the nuclei of IECs (Fig. 1p, q). The qPCR results showed that the expression levels of CD34, kinase insert domain receptor (Kdr), nitric oxide synthase (Nos)2, and Nos3 in IECs were significantly increased compared with those in BM-MSCs (Fig. 1r). IECs were positively stained for Pecam1 and vWF (Fig. 1s, t). BM-MSC-derived IOBs showed positive alizarin ALP and red S staining (Fig. 1u, v).

### SC-CM promotes IEC proliferation, migration, capillary-like tube formation, and angiogenesis

**Fig. 2:**
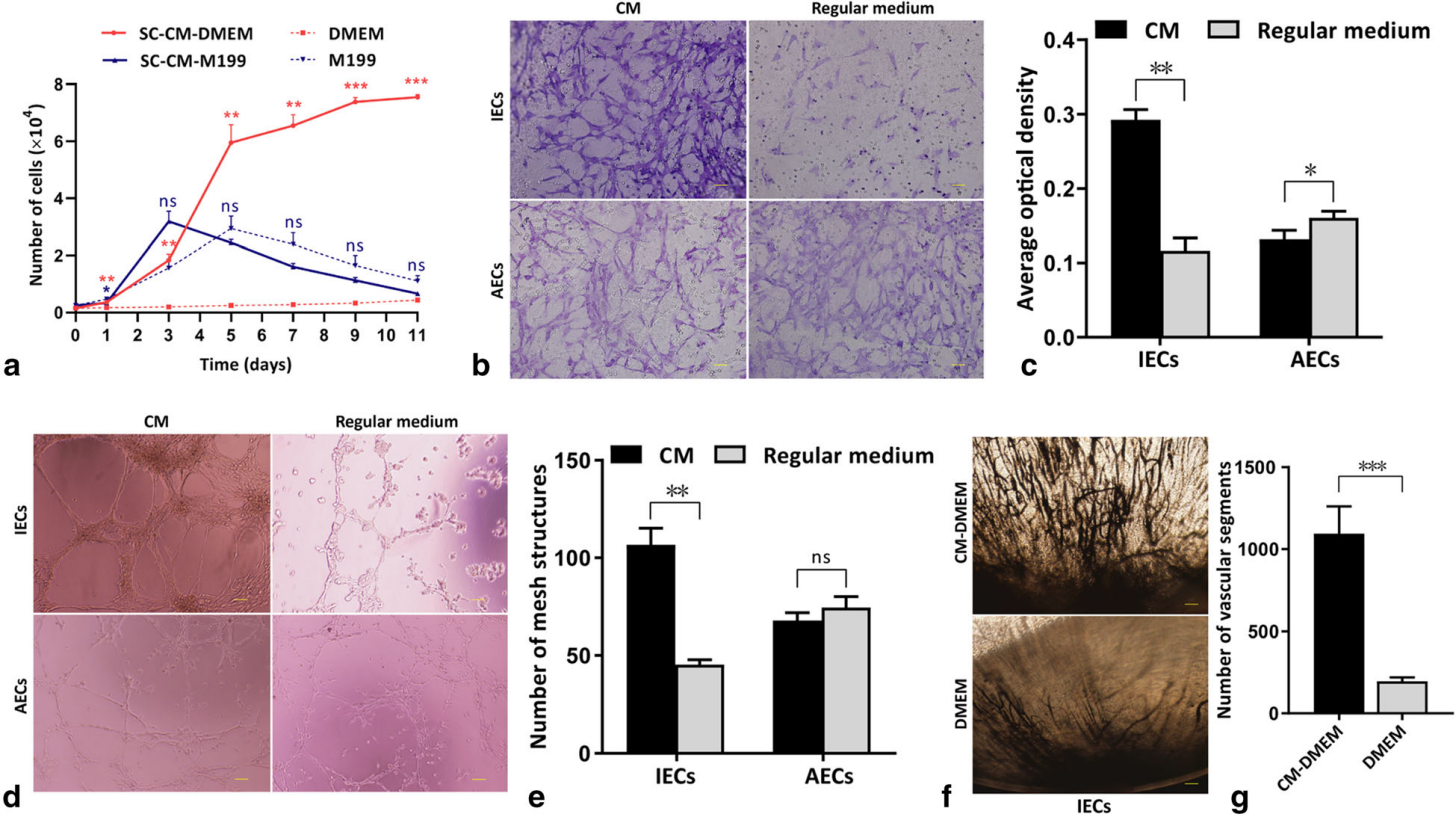
The effects of SC-CM on endothelial cells. **a** Proliferation curves of IECs (red) and AECs (blue) in SC-CM and regular medium. **b** Migrated IECs or AECs in the SC-CM wells and regular medium wells (scale bar = 50 μm). **c** OD values of the eluted fluid of the cell-migrated membranes in each group. **d** Capillary-like tube structures formed by IECs or AECs in Matrigel of SC-CM wells and regular medium wells (scale bar = 100). **e** Capillary-like mesh structure counting of each group. **f** New vessels formed by IECs perfused with ink in the rat corneas of each group (scale bar = 100). **g** Vascular segment counting of the new vessels formed by IECs in rat corneas of each group

The number of IECs cultured in SC-CM-DMEM was significantly higher than that in the control group at each time point. The number of AECs cultured in SC-CM-M199 was significantly lower than that of the control group at 1 day, and no significant difference was shown after 3 days (Fig. 2a). There were more migrated IECs in the SC-CM-DMEM wells than in the DMEM wells, while relatively less migrated AECs in the SC-CM-M199 wells than in the M199 wells (Fig. 2b). The OD values of the eluted fluid illustrated the same result (Fig. 2c). More capillary-like tube structures were found in the CM wells than in the control wells for IECs, while no significant difference was shown for AECs (Fig. 2d), as shown by capillary-like tube structure counting (Fig. 2e). More vessels formed by IECs and more vascular segment counting were observed in the corneas of the SC-CM-DMEM group than in the corneas of the control group on the 12th day (Fig. 2f, g).

### SCs and IECs interact in a coculture system

**Fig. 3:**
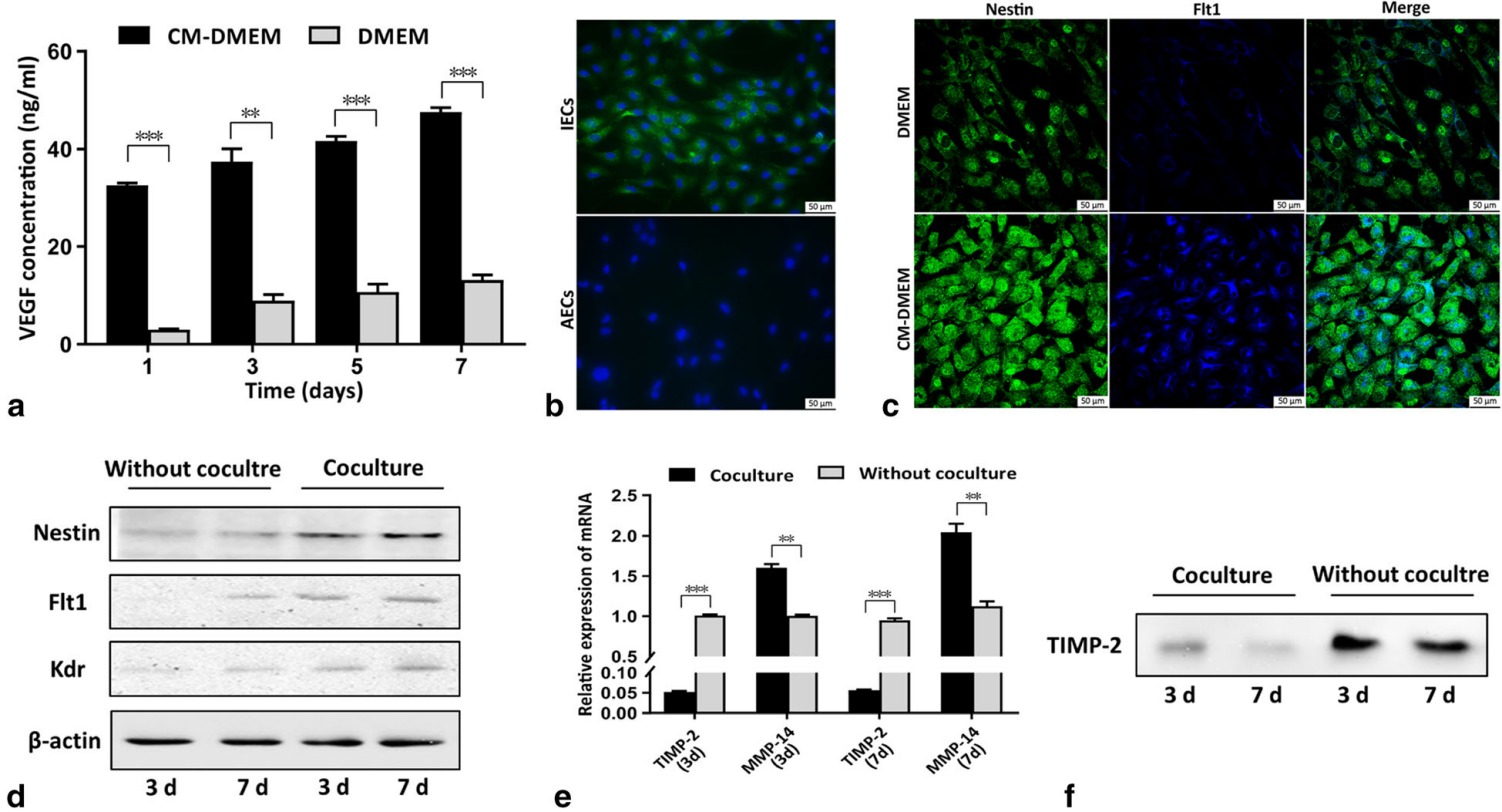
The interaction of SCs and endothelial cells. **a** VEGF secretion levels in the supernatants in each group as determined by ELISA. **b** Nestin expression in IECs and AECs by immunofluorescence staining. **c** Nestin and Flt1 expression in IECs in SC-CM-DMEM or DMEM by double immunofluorescence staining. **d** Nestin, Flt1, and Kdr expression levels in IECs with or without coculture at days 3 and 7 by WB. **e** TIMP-2 and MMP-14 expression levels in SCs cocultured with IECs and without coculture by qPCR. **f** Secreted TIMP-2 expression in the supernatants of the coculture system and the control wells

First, the VEGF concentration in SC-CM-DMEM was detected, and the detected value (499.85 pg/ml in SC-CM-DMEM) confirmed that SCs secreted VEGF. The VEGF concentration in the supernatant during the coculture process was also detected, and the results showed that the VEGF concentration secreted only by IECs in the SC-CM wells increased successively at each time point and was higher significantly than the control group at each time point (Fig. 3a).

We compared nestin expression in IECs and AECs, and the results showed that IECs expressed nestin extensively, while AECs barely expressed nestin (Fig. 3b). Double immunofluorescence staining showed that nestin and Flt1 were simultaneously expressed more strongly in IECs in SC-CM-DMEM wells than in the DMEM wells (Fig. 3c). To further investigate the interaction between SCs and IECs, we detected nestin and VEGF receptor expression levels in IECs and TIMP-2 expression levels in SCs in the coculture system. The WB results showed that the expression levels of nestin, Flt1, and Kdr were significantly higher in IECs cocultured with SCs than in IECs cultured without SCs and revealed an increasing trend as the time in coculture increased (Fig. 3d). The qPCR results showed that the expression level of TIMP-2 in SCs cocultured with IECs was significantly lower than the control wells at days 3 and 7, while matrix metalloproteinase (MMP)-14 expression was significantly higher during the process of coculture (Fig. 3e). The results of secreted TIMP-2 expression in the supernatants showed a decreasing trend when SCs were cocultured with IECs, and the secreted TIMP-2 expression level was significantly lower in the supernatants of the SC and IEC coculture system than without coculture (Fig. 3f).

### Implantation of scaffolds with labeled cells

**Fig. 4:**
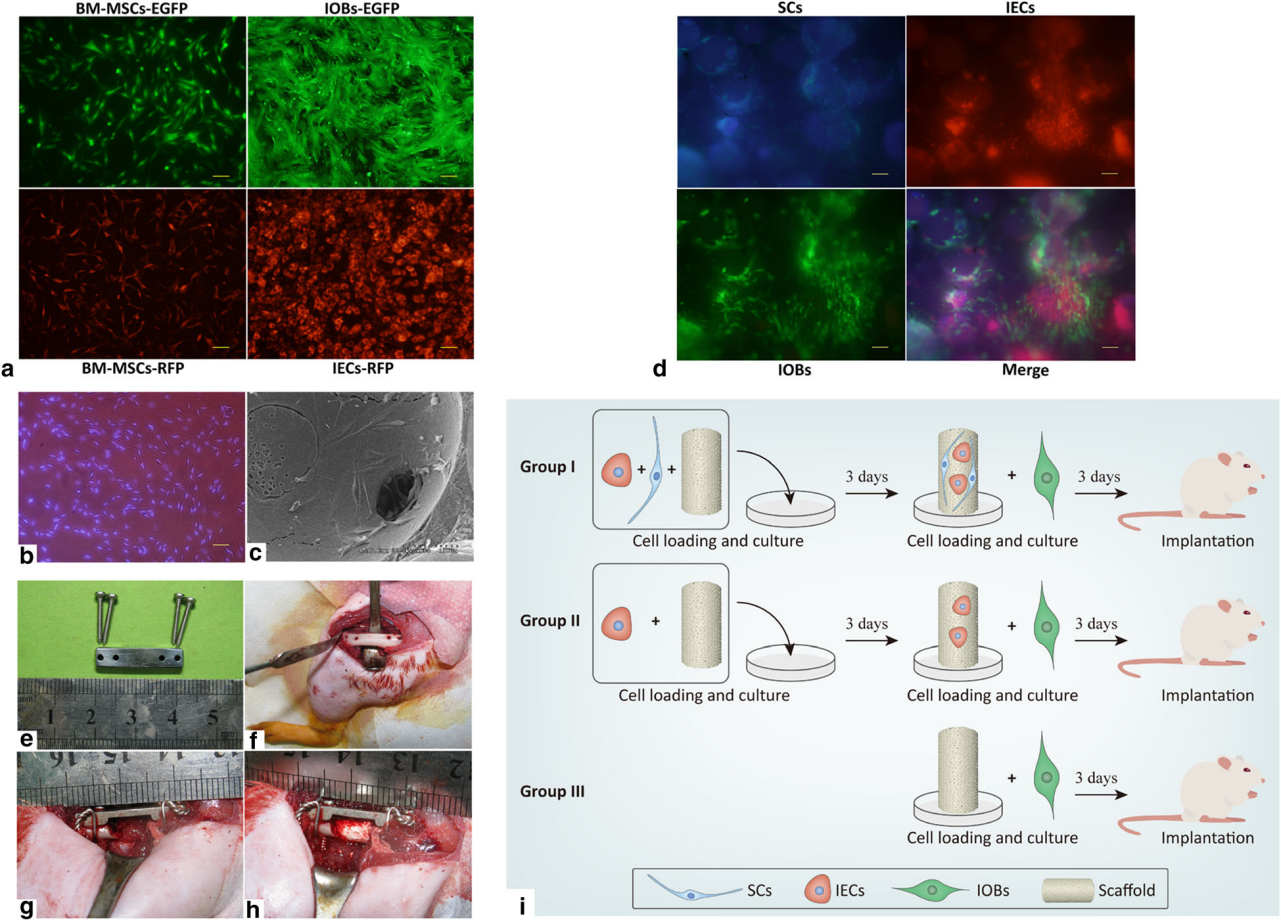
Prevascularized scaffolds loaded with labeled cells were applied in rat models. **a** BM-MSCs, IOBs expressing EGFP, and IECs expressing RFP (scale bar = 100). **b** SCs labeled with Hoechst 33342 (scale bar = 100). **c** SCs, IECs, and IOBs adhered and grew on β-TCP scaffolds observed by SEM at day 6 post-seeding. **d** Labeled SCs, IOBs-EGFP, and IECs-RFP grew on the β-TCP scaffolds to make prevascularized TEBGs (scale bar = 100) at day 6 post-seeding. **e** The internal fixation with steel plate applied in rat operations. **f** Femur exposed and drilled for internal fixation. **g** Six-millimeter-long defect of the middle femur and the steel plate implanted for internal fixation. **h** Prevascularized TEBG implanted into the femur defect. **i** The design of the time schedule and group setting during the experiment

To evaluate the effect of scaffold extract fluid on cell viability, we detected BM-MSC proliferation in 9 days, and no significant difference was found between extract fluid and regular DMEM (Supplementary Fig. 1a). After the MTS reagent was injected into the scaffold seeded with BM-MSCs, the deposition of blue-purple formazan verified the cell viability on the scaffold (Supplementary Fig. 1b, c). To observe the attachment and growth of cells loaded on scaffolds, we labeled BM-MSCs with EGFP and RFP and induced the labeled BM-MSCs to differentiate into IOBs-EGFP and IECs-RFP (Fig. 4a). The nuclei of SCs were labeled with Hoechst 33342 (Fig. 4b). Scanning electron microscopy (SEM) revealed that the seeded cells adhered well to the scaffolds (Fig. 4c). The labeled SCs, IOBs-EGFP, and IECs-RFP grew well on the scaffolds, as observed under a fluorescent inverted microscope (Fig. 4d). The cell-loaded scaffolds were successfully implanted into the femoral defects of rats, and the internal fixations were placed firmly (Fig. 4e–h). The procedures and groups of the experimental design are displayed in Fig. 4i.

### Radiological assessment

**Fig. 5:**
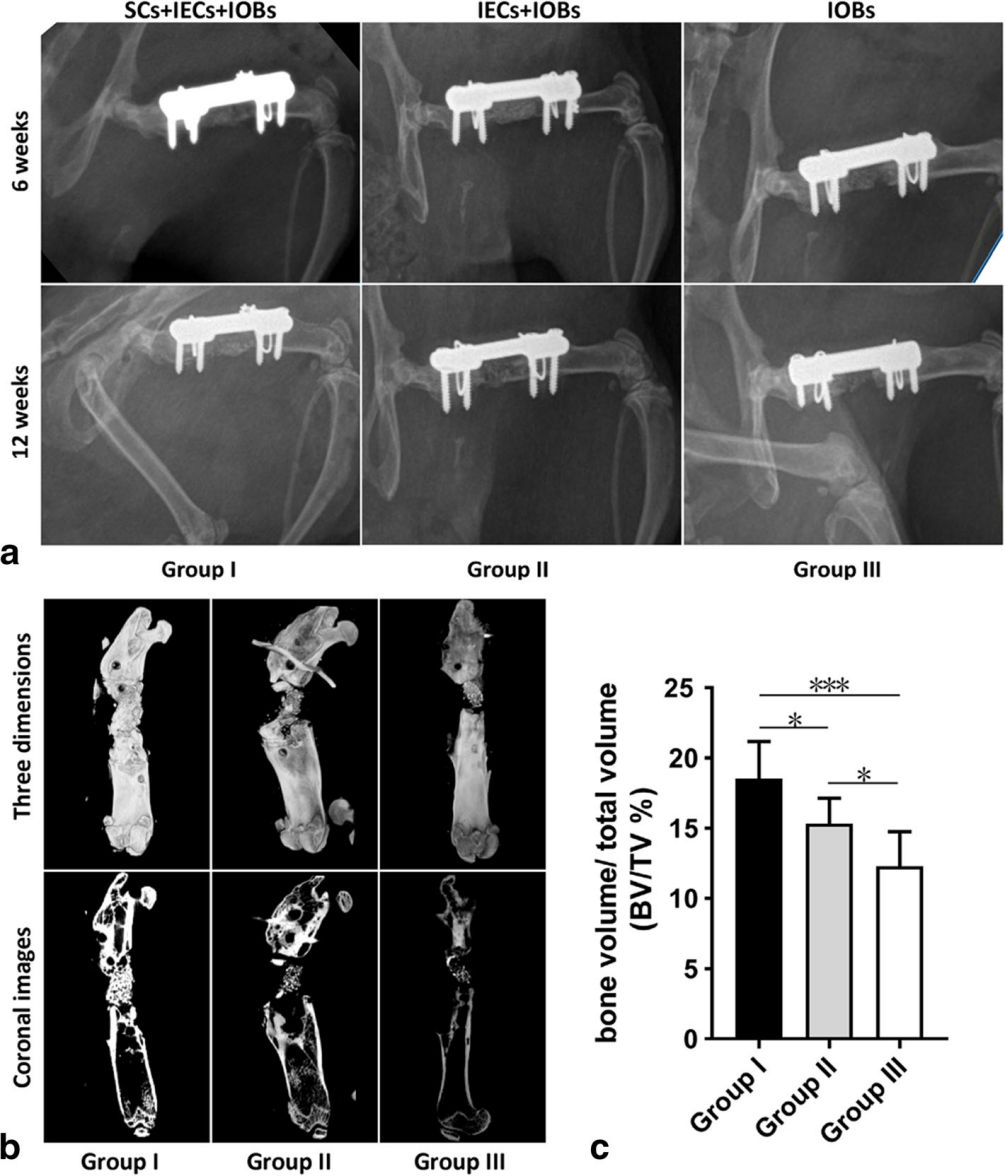
Radiological assessment of TEBGs in vivo. **a** X-ray images after operation 6 weeks and 12 weeks of the three groups. **b, c** The micro-CT images of the three groups (**b**) and the comparison of BV/TV % values (**c**) after removing the internal fixations at 12 weeks postoperatively

At 6 weeks, the X-ray results showed that the bone defects of the rats in each group had not completely healed after the operation. In group I, the TEBG material was absorbed, the internal density was uneven, and a small number of bony calluses formed on the surface of the TEBG. In groups II and III, the density of the TEBG material was significantly different from the density of the surrounding bone, the fracture line was still clearly visible, and there was no bony callus formation. Twelve weeks after the operation, X-ray imaging showed that the bone defects of the rats in group I had healed and that the fracture line had disappeared. Bony calluses were evenly distributed at the defect site and bridged the fracture ends. The bone defects of rats in group II had partially healed, and the fracture line was obscure and showed some bony callus formation. In group III, the bone defect had not healed, and the fracture line was still clear without visible bony callus formation (Fig. 5a).

Micro-CT at postoperative 12 weeks more intuitively showed the difference in TEBG osteogenesis in each group in three dimensions and coronal images (Fig. 5b). The BV/TV % values of the rats in group I were significantly higher than those of the other two groups, and group III showed the lowest value of the three groups (Fig. 5c).

### Histological assessment

**Fig. 6:**
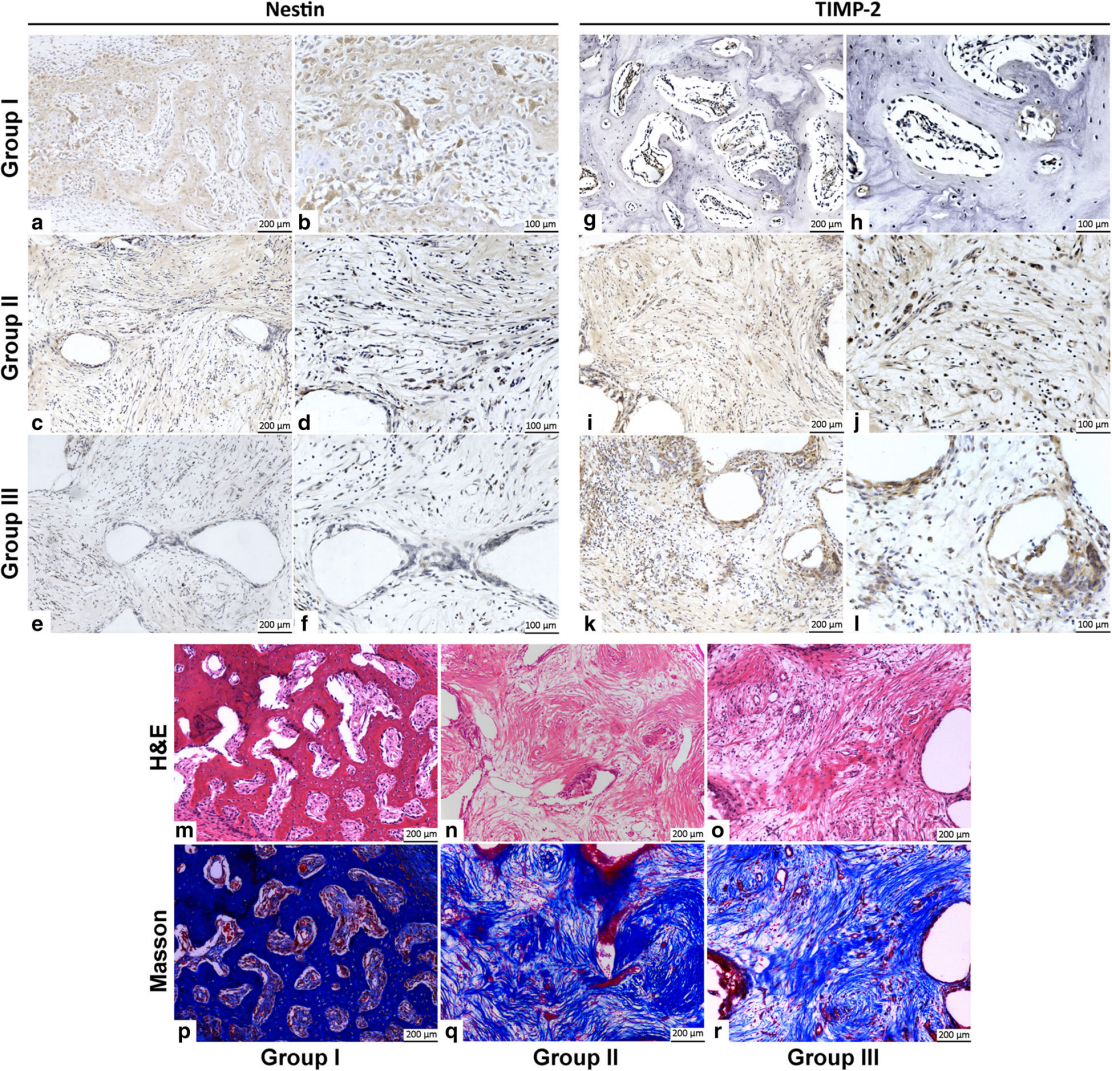
Histological assessment of TEBGs in vivo. **a–f** IHC staining of nestin at 12 weeks in groups I (**a**, **b**), II (**c**, **d**), and III (**e**, **f**). **g–l** IHC staining of TIMP-2 at 12 weeks in groups I (**g**, **h**), II (**I**, **J**), and III (**k**, **l**). **m–r** H&E and Masson staining at 12 weeks in groups I (**m**, **p**), II (**n**, **q**), and III (**o**, **r**)

For IHC staining, in group I, the nestin high-expression areas were significantly more than the other two groups (Fig. 6a, b), while TIMP-2 expression areas were significantly less (Fig. 6g, h). Group II showed sporadic and individual nestin expression cells (Fig. 6c, d) and relatively more TIMP-2 expression areas (Fig. 6i, j). In the areas of new bone formation and growth in group III, almost no nestin expression was observed (Fig. 6e, f), but obvious and more TIMP-2 expression was visible (Fig. 6k, l).

At 12 weeks, in group I, most of the scaffolds degraded and regenerated woven bone formed in the implanted areas. Thickened trabecular bones grew in some areas where Masson staining showed obvious bone growth (Fig. 6m, p). Group II showed that some of the scaffolds degraded and collagen fibrous tissue grew inside the scaffolds, where a small amount of new bone could be observed through H&E and Masson staining (Fig. 6n, q). Group III showed similar histological changes to group II, while fewer scaffolds were absorbed and new bone growth was observed (Fig. 6o, r).

**Fig. 7:**
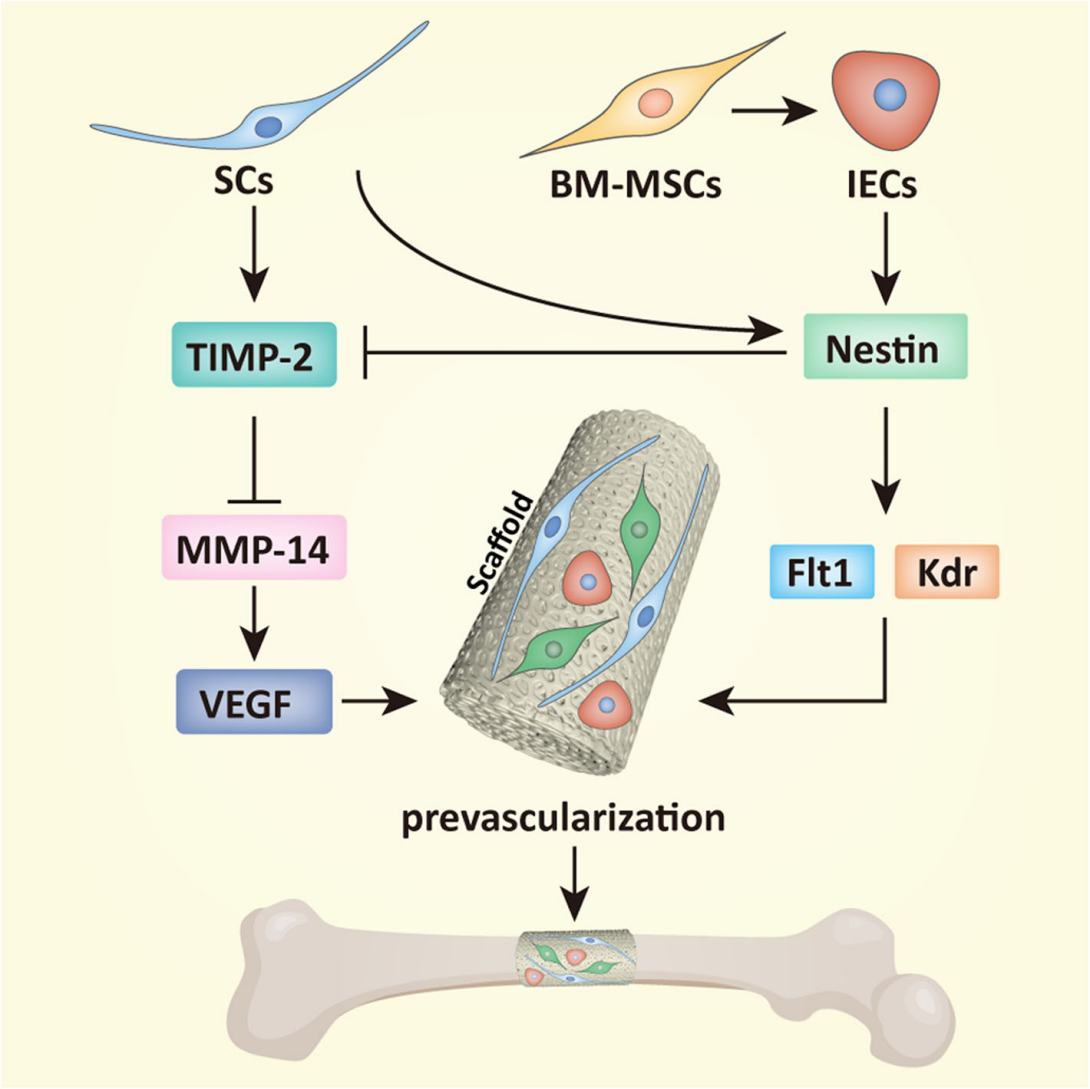
A schematic diagram of the interactions between SCs and endothelial cells in TEBG. SCs promoted nestin and VEGF receptor expression of BM-MSC-derived IECs, and IECs inhibited TIMP-2 secretion of SCs by autologous nestin expression to promote activity of MMP-14 and VEGF, eventually efficiently induced prevascularization and osteogenesis of TEBG

In summary, the results of this study further revealed that SCs promoted the angiogenesis- and vascularization-related activities of BM-MSC-derived IECs by regulating nestin and TIMP-2, in which MMP-14 and VEGF and VEGF receptors involved, whereas IECs inhibited TIMP-2 secretion of SCs by autologous nestin expression, eventually efficiently induced prevascularization and osteogenesis of TEBG (Fig. 7).

## Discussion

The bone is fully vascularized and innervated where blood vessels and nerve fibers closely interact [29]. Blood vessels and nerve fibers, which exist throughout the entire bone, including the periosteum, bone marrow, and mineralized parts of the bone, are involved in bone formation and development [29, 30]. Vascular and neural involvement is the basis for the construction of normal functional bone tissue during TEBG osteogenesis. To date, numerous studies have focused on the vascularization of TEB, but far less is known regarding the neurotization of TEB. Therefore, our group proposed constructing TEB that is simultaneously highly vascularized and neurotized [31]. We made some efforts to construct neurovascularized TEB, including implanting sensory nerve tracts through microsurgical techniques, which is a feasible method for constructing neurotized TEB [16–18, 31]. In this study, we revealed a correlation between neural and vascular seed cells in the construction of TEBs and verified that SCs and IECs promote the osteogenesis of prevascularized TEBGs.

BM-MSCs can be induced to differentiate into endothelial cells by some cell factors, including VEGF and basic fibroblast growth factor (bFGF) [32, 33]. In this study, we used BM-MSCs as an alternative source of IECs by using EGM-2 SingleQuots, which includes VEGF, bFGF, insulin-like growth factor (IGF), and epidermal growth factor (EGF). The results of cell identification showed that the morphology of IECs, including the appearance of the cells and their organelles, was changed and that the expression of the endothelial cell-specific genes vWF and Pecam1 and Nos2 and Nos3, which are genes related to endothelial cell function, was significantly increased in IECs compared to BM-MSCs. IECs formed capillary-like tube structures and underwent angiogenesis in subsequent experiments. These findings confirm that IECs can be considered sources of vasculature with effective biochemical functions for bone tissue engineering.

As the component cells of the myelin sheath, SCs are commonly used as seed cells in neural tissue engineering [34]. SCs not only improve axonal regeneration but also secrete VEGF to play a protective role in axonal growth [35]. In addition, sensory nerves or SCs can induce arterial marker expression in embryonic endothelial cells by locally secreting VEGF [36]. SCs have also been shown to promote human umbilical vein cell (HUVEC) migration through VEGF [37]. Transplantation of a combination of microencapsulated SCs and BM-MSCs has been verified to augment angiogenesis by increasing local VEGF levels [38]. In the present study, we found that SC-CM promoted IEC proliferation, migration, capillary-like tube formation, and angiogenesis. As shown by ELISA, VEGF was present in SC-CM at a relatively high concentration. The VEGF concentration also gradually increased with time in SC and IEC cocultures. VEGF is well known to be a key molecule for the proliferation, migration, and angiogenesis of endothelial cells [39]. Thus, VEGF was speculated to be an important factor through which SC-CM promoted IEC proliferation, migration, and angiogenesis in this study.

However, some previous studies have confirmed that SC-CM inhibits AEC angiogenesis in vitro and in vivo through TIMP-2 [22, 23]. We examined the effect of SC-CM on AECs and found that SC-CM did not significantly promote AEC proliferation, migration, or capillary-like tube formation. We further found that IECs expressed nestin extensively, while AECs barely expressed nestin. Nestin has been characterized as a potent marker of angiogenesis and neovascularization because it is expressed in endothelial cells with stem-like characteristics but not in mature endothelial cells [40]. Moreover, it has been confirmed that nestin expression is induced in capillary endothelial cells through the VEGF signaling pathway [41]. VEGF facilitates nestin expression to induce endothelial cell migration and angiogenesis by acting on VEGF receptors, including Flt1 [42, 43]. The results of qPCR and immunofluorescence staining in our study also showed a significant increase in nestin and Flt1 expression in IECs upon stimulation with SCs. Therefore, the difference in the effect of SCs on endothelial cells is due, at least in part, to the differential expression of nestin in endothelial cells of different origins. It can be inferred that SC-derived VEGF can promote IEC proliferation, migration, and angiogenesis in IECs through nestin.

Angiogenesis is thought to be regulated by the balance between inducers and inhibitors within a given microenvironment [44]. As a member of the TIMP family, TIMP-2 has been shown to suppress angiogenesis to inhibit the activities of MMPs [23, 45]. In a noncontact coculture system, SCs affect IECs by secreting exogenous molecules, and IECs also affect SCs. Our results showed that TIMP-2 expression was inhibited in SCs cocultured with IECs and that this inhibition persisted throughout the duration of coculture. To compare the effects of endothelial cells of different origins on SCs, we also analyzed SCs cocultured with AECs. However, TIMP-2 was not observed to have an obvious inhibitory effect. MSCs have been confirmed to inhibit TIMP-2 generation and support MMP release by cocultured cardiac fibroblasts or skeletal muscle cells [46, 47]. In our experiment, qPCR showed that MMP-14 expression was slightly enhanced in SCs cocultured with IECs. TIMP-2 is known to extensively inhibit angiogenesis by binding to MMP-14 and other MMPs [45]. Thus, we speculated that IECs retain some of the features and functions of BM-MSCs, allowing them to inhibit the secretion and effect of exogenous TIMP-2 to subsequently enhance angiogenesis and vascularization.

We used a large segmental femoral defect model in rats in this study. Critical-sized defects (CSDs) are commonly 1.5 to 2 times the diameter of long tubular bones [48]. We created a 6-mm-long femoral defect in 10-week-old male SD rats, which had a middle femur cross-section diameter of 25 to 30 mm. Ten-week-old SD rats have a relatively low growth rate of long bones, which allowed the longitudinal stability of the femur and the consistency of the long bone defect during the observation period.

Prevascularization involves vascularization of the scaffold in vitro or in vivo and is a promising method for enhancing the vascularization of tissue constructs upon implantation [49]. We previously performed prevascularization with the insertion of the vascular bundle into scaffolds, which can promote the recruitment of endogenous cells and collagen deposition to establish fast-acting angiogenesis and osteogenesis [50, 51]. Prevascularization also induced by endothelial cells in vitro, which can form original vessel structures in vitro to achieve better vascularization and osteogenic efficacy, has been used in TEB construction before implantation [52, 53]. Our experiments also adopted this method; IECs and SCs were preseeded and cultured before IOBs were seeded and implanted into femoral defects in rats. SCs have dual angiogenic and neurotrophic effects during prevascularization and TEB construction. Moreover, SCs have been shown to enhance osteoblast proliferation and differentiation [54]. Thus, SCs probably have three effects, namely, promoting vascularization, underlying neurotization, and promoting osteogenesis, in TEB construction. In the present study, we observed the effects of SCs on vascularization and osteogenesis in a rat model through radiological and histological assessments and verified that nestin expression increased and TIMP-2 expression decreased due to the involvement of SCs in vivo. It is true that the relationship between vascularization, neurotization, and osteogenesis is complex due to the number of factors involved. Indeed, SCs are only a basic factor required for the neurotization of TEB; further investigation of other factors associated with nerves, including sensory neurons, which are related to osteoblasts and SCs, and related mechanisms underlying TEB are urgently needed [55].

## Conclusions

A variety of cells are involved in the process of osteogenesis and functionalization of TEBGs. Exploring the interaction between seed cells is important to find ways to promote bone regeneration. In this study, we revealed that SCs can promote the proliferation, migration, and angiogenesis of BM-MSC-derived IECs by regulating nestin and autologous TIMP-2. Consequently, SCs promote TEBG vascularization and osteogenesis to repair rat femoral large segmental defects. Meanwhile, our results provide preliminary cytological evidence to support the neurotization of TEB.

## Supplementary Information

**Additional file 1: Supplementary Fig. 1.** Cell viability assessment on scaffolds. (A) BM-MSC proliferation in scaffold extract fluid or regular DMEM in 9 days. (B, C) Deposition of blue-purple formazan in the scaffold after injecting MTS reagent into the scaffold loaded with BM-MSCs.

## Data Availability

All supporting data and materials are included in the article.

## Abbreviations

AECs: Aortic endothelial cells
ALP: Alkaline phosphatase
Ara-C: Cytosine-B-arabinoside hydrochloride
BDNF: Brain-derived neurotrophic factor
bFGF: Basic fibroblast growth factor
BM-MSCs: Bone marrow-derived mesenchymal stem cells
BSA: Bovine serum albumin
BV/TV: Bone volume/total volume
β-TCP: β-Tricalcium phosphate
CSDs: Critical-sized defects
CM: Conditioned medium
DAPI: 4′,6-Diamidino-2-phenylindole
DMEM: Dulbecco’s modified Eagle’s medium
ECGS: Endothelial Cell Growth Supplement
EGF: Epidermal growth factor
EGFP: Enhanced green fluorescent protein
EGM-2 SingleQuots: Endothelial Growth Medium-2 supplements and factors
ELISA: Enzyme-linked immunosorbent assay
FBS: Fetal bovine serum
GAP43: Growth-associated protein-43
GAPDH: Glyceraldehyde-3-phosphate dehydrogenase
GFAP: Glial fibrillary acidic protein
HUVEC: Human umbilical vein cell
IECs: Induced endothelial cells
IGF: Insulin-like growth factor
IHC: Immunohistochemistry
IOBs: Induced osteoblasts
Kdr: Kinase insert domain receptor
M199: Medium 199
MMP: Matrix metalloproteinase
MPZ: Myelin protein zero
MTS: 3-(4,5-dimethylthiazol-2-yl)-5-(3-carboxymethoxyphenyl)-2-(4-sulfophenyl)-2H-tetrazolium
NGF: Nerve growth factor
Nos: Nitric oxide synthase
OD: Optical density
PBS: Phosphate-buffered saline
qPCR: Quantitative reverse transcription polymerase chain reaction
RFP: Red fluorescent protein
RIPA: Radioimmunoprecipitation assay
SCs: Schwann cells
SD: Sprague-Dawley
SDS: Sodium dodecyl sulfate
SEM: Scanning electron microscopy
SOX10: SRY-related HMG-box 10
TEB: Tissue-engineered bone
TEBGs: Tissue-engineered bone grafts
TEM: Transmission electron microscopy
TIMP-2: Tissue inhibitor of metalloproteinase-2
VEGF: Vascular endothelial growth factor
vWF: von Willebrand factor
WB: Western blot
