# Supplementary figures and images for: Schwann cells promote prevascularization and osteogenesis of tissue-engineered bone via bone marrow mesenchymal stem cell-derived endothelial cells
Source: Stem Cell Res Ther. 2021 Jul 7;12:382. doi: 10.1186/s13287-021-02433-3 (PMC8261922; doi:10.1186/s13287-021-02433-3)

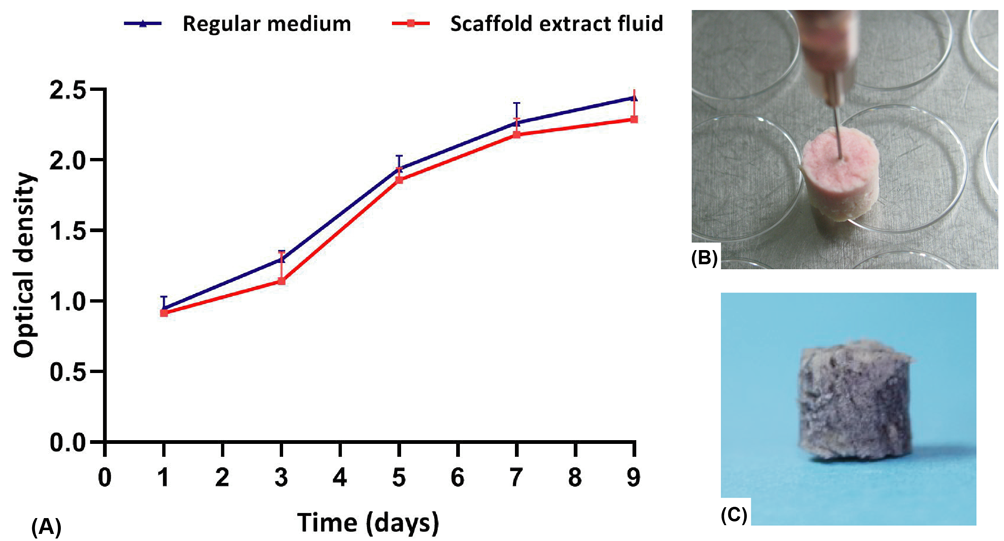

Supplement: Supplementary file 1 — Additional file 1: Supplementary Fig. 1. Cell viability assessment on scaffolds. (A) BM-MSC proliferation in scaffold extract fluid or regular DMEM in 9 days. (B, C) Deposition of blue-purple formazan in the scaffold after injecting MTS reagent into the scaffold loaded with BM-MSCs. [file 13287_2021_2433_MOESM1_ESM.tif]
